# Acute symptoms related to air pollution in urban areas: a study protocol

**DOI:** 10.1186/1471-2458-6-218

**Published:** 2006-08-25

**Authors:** Masud Yunesian, Fariba Asghari, Javad Homayoun Vash, Mohammad Hossein Forouzanfar, Dariush Farhud

**Affiliations:** 1 School of Public Health & Centre for Environmental Research, Tehran University of Medical Sciences; Poursina Street, Keshavarz Boulevard, P.O. Box 14155-6446, Tehran, Iran; 2 Medical Ethics Research Center, Tehran University of Medical Sciences, Tehran, Iran; 3 Department of Epidemiology and Biostatistics, School of Public Health, Tehran University of Medical Sciences, Tehran, Iran; 4 Department of Human Genetics, School of Medicine, Tehran University of Medical Sciences, Tehran, Iran

## Abstract

**Background:**

The harmful effects of urban air pollution on general population in terms of annoying symptoms are not adequately evaluated. This is in contrast to the hospital admissions and short term mortality. The present study protocol is designed to assess the association between the level of exposure to certain ambient air pollutants and a wide range of relevant symptoms. Awareness of the impact of pollution on the population at large will make our estimates of the pertinent covert burden imposed on the society more accurate.

**Methods/design:**

A cross sectional study with spatial analysis for the addresses of the participants was conducted. Data were collected via telephone interviews administered to a representative sample of civilians over age four in the city. Households were selected using random digit dialling procedures and randomization within each household was also performed to select the person to be interviewed. Levels of exposure are quantified by extrapolating the addresses of the study population over the air pollution matrix of the city at the time of the interview and also for different lag times. This information system uses the data from multiple air pollution monitoring stations in conjunction with meteorological data. General linear models are applied for statistical analysis.

**Discussion:**

The important limitations of cross-sectional studies on acute effects of air pollution are personal confounders and measurement error for exposure. A wide range of confounders in this study are controlled for in the statistical analysis. Exposure error may be minimised by employing a validated geographical information system that provides accurate estimates and getting detailed information on locations of individual participants during the day. The widespread operation of open air conditioning systems in the target urban area which brings about excellent mixing of the outdoor and indoor air increases the validity of outdoor pollutants levels that are taken as exposure levels.

## Background

Air pollution is a major health problem globally. Industrialization and ongoing development of large urban areas in many countries are posing increasing number of people to potential hazards of air pollution. There are reports of adverse effects of carbon monoxide, sulphur dioxide, nitrogen dioxide, ozone, and particulate matter with aerodynamic diameter less than 10 micrometer (PM10) on hospital admissions for cardiovascular [1,2] and respiratory [3,4] diseases as well as daily mortality [5,6]. On the other hand, the effects of the ambient air pollution on general population causing minor complaints not resulting in medical consultations is not properly investigated particularly in developing countries. The reports in the literature mainly concentrate on the respiratory symptoms in young children [7-9]. The current study is designed to evaluate the relationship between air pollution and a wide range of relevant symptoms in an urban population to discover the associations between each symptom and any of the five pollutants mentioned above. The findings will help us realize the scope of the problem and more realistically quantify the air pollution related losses.

The burden of air pollution on health system is generally underestimated. The unseen part of the problem in the form of annoying and occasionally debilitating symptoms in the general population of the urban areas is not adequately studied. Symptoms due to air pollution are generally neither consulted nor registered in medical records, especially in developing countries; therefore, the costs of research on this subject is significantly higher than recorded hospital admissions and mortalities.

## Methods/design

### Aim and design

This study investigates the association between a range of air pollutants and a set of symptoms not necessarily resulting in hospital admissions or practitioner consultations in an urban area. A cross sectional analysis is made to determine the association between personal levels of exposure to different pollutants and presence or absence of certain complaints. Exposure and outcome data are gathered during a ten month period allowing for emergence of a wide range of potential confounding factors such as meteorological variables that are taken into account in the statistical analysis.

### Hypothesis

A set of complaints that can be referred to air pollution was identified by literature review by two of the authors and the primary lists were matched and consensus reached by discussion of all authors over the final set of symptoms to be surveyed. We decided to investigate the association between the levels of carbon monoxide (mean 24 hour CO level measured by spectrophotometer), sulphur dioxide (mean 24 hour SO2 level measured by UV fluorescence), nitrogen dioxide (mean 24 hour NO2 level measured by chemiluminescence), ozone (maximum moving average for 8 hours concentration of O3 measured by UV photometry), and particulate matter less than 10 micron in aerodynamic diameter (mean 24 hour concentration of PM10 measured by gravimetric) on one hand and the following symptoms on the other: wheeze, breathlessness (dyspnoea), cough, phlegm, chest tightness, palpitations, nausea, headache, eye irritation and sore throat. As the level of ozone changes significantly during the day and the symptoms related to ozone appear within a short interval after exposure, the maximum eight hour concentration is considered as the independent variable; unlike other pollutants which are averaged on daily basis. Because of the possible delayed effects of the pollutants all combinations for the levels of the pollutants in the last 72 hours are tried when looking for a regression model that best fits the available data. We considered the following as potential confounding variables: mean daily temperature of the city, relative humidity, day of week, season, holiday effect (three categories of holiday, the day after holiday, and others), history of smoking (categorized as: has never been smoker, used to smoke, passive smoker, occasional smoker, and habitual smoker), exposure hours (the hours of exposure to outdoor air during the day), occupational exposure to air pollutants, home heating appliance, presence of common cold in the person's household, history of asthma in the person or first degree relatives, age, gender, educational attainment of the family superintendent, and economic status.

### Setting

This study is designed for the city of Tehran, the largest city of the Middle East with an area of 1500 sq km and a population of 6.7 millions according to the last census (1996) and around 2.6 million cars running in the streets every working day. The study population are residents of the city more than four years of age at the time of the study. Expressive language development in children of this age is complete enough to let them communicate different symptoms reliably.

### Measures of exposure and outcome

City area is divided into square blocks of 500 by 500 meters on the map and the level of pollutants for each of these blocks is estimated from a model incorporating the level of the pollutants measured in the four air quality monitoring stations and also taking into account the meteorological factors of the city at the time. Levels of exposures of the participants to each of the pollutants were extracted from this geographical information system for both home and workplace addresses and the higher value for each pollutant were considered the individual's exposure to that specific pollutant. Outcome measures that are presence or absence of any of the symptoms listed above was asked from participants on the phone by the interviewers.

### Data gathering and quality control

The study population were asked about their symptoms by telephone interview after random digit dialling of one of the landline numbers from the city between 4 PM and 8 PM. A pilot was performed to define the best time for interview in terms of successful contacts and to estimate the time needed to obtain the necessary information from selected member of the household. The mean time for a successful interview was seven minutes but there were some contacts to non-residential buildings and absence of an adult in some households postponed the interview to a later time, as a result the actual number of successful interviews during the 30 hours of pilot was 151. Therefore, five contacts per hour was set as the goal and each interviewer was expected to complete 20 interviews every working day.

**Figure 1:**
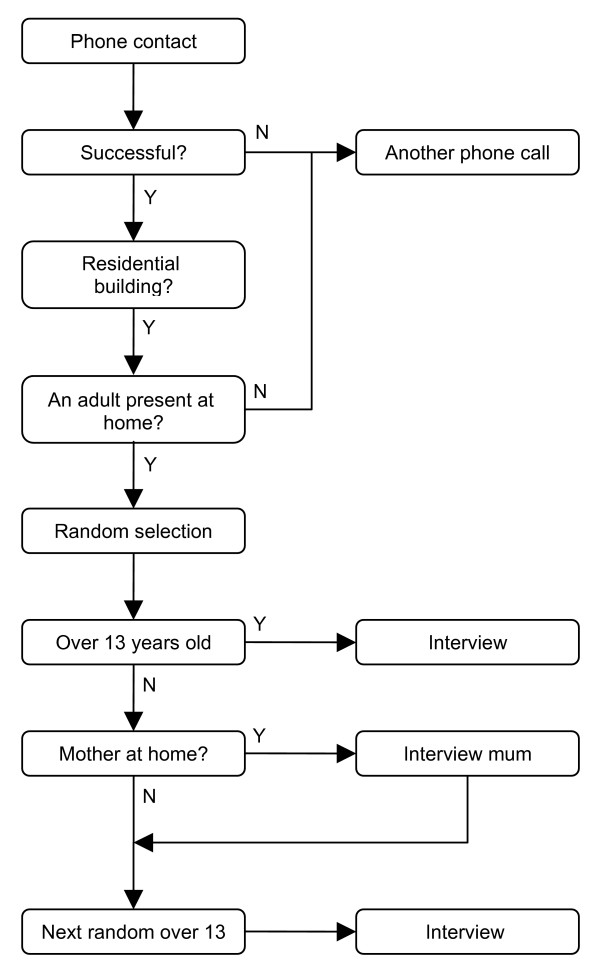
Algorithm used for telephone interview.

Sample population were contacted by random selection without replacement from the exhaustive list of landlines obtained from the telephone service provider of the city thus simple random sampling. The interviewers used to ring the selected lines and talk to one of the adults in the household. They used to introduce themselves and explain the purpose of the study to encourage the person to take part in the survey. If they agreed to participate, the interviewer would select one of the members of the household by asking the birth date of all members older than four years of age and choosing the person whose birthday is closest to interview date. The selected persons answered the questions themselves if they were older than 13 years old, otherwise the mother would help them answer their questions and next random match from the family, older than 13, would be interviewed as well. If nobody answered the phone or there was no adult at home, the same line would be contacted again at the same day. It would be replaced with another random number if it was found idle again (Figure 1).

The interview team was a group of eight interviewers and a supervisor. The interviewers were selected on the basis of their occupational background in research projects and their communication skills. They received training on the method of randomization, the conduct of the interview, and the definition of the terms in the questionnaire. The supervisor was an expert social sciences research assistant and she monitored the interviews for compliance with the protocol and re-interviewed seven percent of the sample of each interviewer in each day and checked the original answers obtained. The maximum acceptable error for each interviewer was considered 15% of the questions. In addition, the executive manager of the research used to contact 10% of the daily sample and checked the accuracy of the data gathering. That is, the performance of the interviewers and the supervisor were checked again and if the problems observed could not be solved by further training the relevant interviewer would be replaced.

The pollutants information matrix of the city identifies the level of pollutants for that day according to the geographical latitude and longitude of the point of interest. Levels of the pollutants were extracted from the matrix for two addresses of home and workplace and the higher reading was considered as individual's exposure to that pollutant. Candidates for the job received training on extracting the longitude and latitude of the sample population addresses from the map and then a team of five clerks were selected on the basis of a similar test performance. A double check method was applied by assigning another clerk to extract geographical data for 25% of the addresses of each clerk. That is, all the addresses were used by two members of the team and the supervisor checked for mismatch and when discovered, the data for that address would be re-extracted.

Data entry was performed by a team of six clerks in SPSS data sheets. A random sample of 100 cases were selected from each clerk's work and checked for three fields of longitude, latitude and telephone number. Up to three errors were acceptable; otherwise the data entry had to be repeated.

### Statistical analysis

Backward stepwise logistic regression is used for analysis. Individual levels of exposure to air pollution are considered as the independent variables and the presence of the complaints as the dependent variable. 95\% confidence interval is calculated for a measure of increase in the frequency of any of the symptoms attributable to one unit increase in the level of each of the pollutants. When assessing the association between the levels of the pollutants with the individual symptoms, exposure to the other pollutants are considered as confounders in conjunction with the other personal and environmental confounding factors mentioned above. To test for autocorrelation, different regression models are analysed and Bootstrap method is implied to estimate the standard error for coefficients of the models.

### Sample size

According to Poisson distribution the sample needed for detecting an increase of 10% in the frequency of the symptoms when the level of pollutant rises one interquartile (IQR, the 75th -25th percentile pollutant values) will be 400 symptomatic cases in each quartile. Consequently, overall 1600 cases will be needed and assuming the prevalence of the least common symptom as 5% the number of successful telephone contacts needed will be 32000.

## Discussion

Health effects of air pollution can be divided in two categories of acute effects resulting from exposure in a short period of time and chronic effects from long term contact. The latter may be assessed by evaluation of the outcomes in cross-sectional surveys comparing areas with different levels of pollutions. Similarly, cohorts from communities with different environmental pollutions may be followed [10].The short term effects of the air pollution, on the other hand, are generally assessed by time-series studies as well as cross sectional and before-after designs [11]. Before-after designs are much like studies on infectious diseases; there is a short interval between exposure and outcome and the source of pollution is clearly determined. Cross-sectional studies focus on the comparison of the populations with different levels of exposure and they resulted in definition of maximum acceptable levels of pollutants in the atmosphere. Time-series studies are especially useful to discover the harmful effects of air pollution in levels lower than the standards [12]. The special advantage of this design is the elimination of personal confounders created by selection bias in the previous studies as in time-series design the study population is compared with itself during a relatively short period of time. On the other hand, this design is not useful for investigating the associations of the chronic conditions with air pollution and is a relatively weak design to suggest cause-effect relationship. Likewise, 'harvesting' is another important limitation of this design. When a noxious element is present for a long period of time those with borderline health conditions will show the symptoms acutely and may get admitted or succumb to death but in the long run this particularly susceptible population may not appear in our symptomatic group and this may result in underestimation of the overall burden [13]. In other words, the acute effect of air pollution will be evident more dramatic than the effects in the long run when the pollutant is present for a prolonged time.

The main limitation of cross-sectional studies on air pollution is the personal confounders in the population. Therefore, numerous variables should be assessed in the survey and controlled for in the statistical analysis. Exposure measurement error, a main limitation of epidemiological studies on environmental effects on human health, readily applies to all types of studies mentioned above [14]. As human exposure to pollutants takes place in a long period of time and in places with different levels of pollution, making accurate estimation of the individual exposure is difficult if not impractical. It is generally believed that the measurements made on outdoor air are acceptable estimates of the population exposure [11] and it may be argued this is particularly applicable to communities with open air conditioning systems that provide excellent mixing of indoor and outdoor air. Outdoor air pollution is especially important in large urban areas particularly in developing countries that have not been as successful as developed nations in controlling the sources of emissions. Taking outdoor levels as a proxy for overall air pollution exposure, we estimated the levels of pollution in addresses of workplace and home that correspond to the places where people spend most of their time and took the higher level as the exposure measure. This method is useful for investigating the acute symptoms resulting from air pollution exposure in contrast to the chronic effects which mostly correspond to the cumulative exposure levels during a long period of time and in different locations.

This study used the data available from multiple air pollution monitoring stations spread throughout the city area and the air pollution matrix enabled us to determine a reasonable estimate of the level of exposure of the participants. This geographical information system is definitely superior to dispersion models that mostly suits the particulate pollutions from certain local sources [8]. Ideally, data from the system should be validated by portable measurement stations and the optimised system with the least possible errors should be implemented for exposure estimates.

Random digit dialling for sampling was biased by more chance for members of small families to be interviewed in comparison with larger families and also more chance for members of households with two or more landlines. Similarly, it may be argued that women will have more chance to appear in the sample as in the time interval between 4 – 8 PM many men are still at work and there are numerous businesses which are predominantly run by men in Tehran. However, this can not affect the findings of the study because people with different levels of exposure were compared in this design and their baseline characteristics were considered as confounders.

Despite widespread use of telephone interview in asking symptoms of patients in different settings the accuracy of the method is largely unknown. There is evidence that people reliably report their symptoms on the phone but they may not be able to assess and report relevant physical signs [15]. It is also believed that the method may be particularly biased in evaluation of the musculoskeletal symptoms [16]. Therefore, it may be claimed that the set of symptoms targeted in this study are reliably assessed by telephone interview.

## Competing interests

The author(s) declare that they have no competing interests.

## Authors' contributions

MY suggested the conception, all authors (MY, FA, JHV, MHF, DF) participated in design of the study, FA supervised data gathering, MHF analysed the data and all authors interpreted the results. JHV drafted the manuscript and it was critically revised by all authors and they have given final approval of the version to be published.

## Pre-publication history

The pre-publication history for this paper can be accessed here:
